# Imaging cervical cytology with scanning near-field optical microscopy (SNOM) coupled with an IR-FEL

**DOI:** 10.1038/srep29494

**Published:** 2016-07-12

**Authors:** Diane E. Halliwell, Camilo L. M. Morais, Kássio M. G. Lima, Julio Trevisan, Michele R. F. Siggel-King, Tim Craig, James Ingham, David S. Martin, Kelly A. Heys, Maria Kyrgiou, Anita Mitra, Evangelos Paraskevaidis, Georgios Theophilou, Pierre L. Martin-Hirsch, Antonio Cricenti, Marco Luce, Peter Weightman, Francis L. Martin

**Affiliations:** 1 Centre for Biophotonics, LEC, Lancaster University, Lancaster, UK; 2 Biological Chemistry and Chemometrics, Institute of Chemistry, Federal University of Rio Grande do Norte, Natal 59072-970, RN, Brazil; 3 Institute of Astronomy, Geophysics and Atmospheric Sciences, University of São Paulo, Brazil; 4 Department of Physics, University of Liverpool, Oliver Lodge Building, Liverpool, UK; 5 Accelerator Science and Technology Centre (ASTEC), STFC Daresbury Laboratory, UK; 6 Institute of Reproductive and Developmental Biology, Department of Surgery & Cancer, Faculty of Medicine, Imperial College, London, UK; 7 West London Gynaecological Cancer Centre, Imperial College NHS Healthcare, London, UK; 8 Department of Obstetrics and Gynaecology, University of Ioannina, Ioannina, Greece; 9 St James Hospital, Leeds, West Yorkshire, UK; 10 Department of Obstetrics and Gynaecology, Lancashire Teaching Hospitals NHS Trust Foundation, Preston, UK; 11 Istituto di Struttura della Materia, CNR, via del Fosso del Cavaliere 100, Rome, Italy; 12 School of Pharmacy and Biomedical Sciences, University of Central Lancashire, Preston, UK

## Abstract

Cervical cancer remains a major cause of morbidity and mortality among women, especially in the developing world. Increased synthesis of proteins, lipids and nucleic acids is a pre-condition for the rapid proliferation of cancer cells. We show that scanning near-field optical microscopy, in combination with an infrared free electron laser (SNOM-IR-FEL), is able to distinguish between normal and squamous low-grade and high-grade dyskaryosis, and between normal and mixed squamous/glandular pre-invasive and adenocarcinoma cervical lesions, at designated wavelengths associated with DNA, Amide I/II and lipids. These findings evidence the promise of the SNOM-IR-FEL technique in obtaining chemical information relevant to the detection of cervical cell abnormalities and cancer diagnosis at spatial resolutions below the diffraction limit (≥0.2 μm). We compare these results with analyses following attenuated total reflection Fourier-transform infrared (ATR-FTIR) spectroscopy; although this latter approach has been demonstrated to detect underlying cervical atypia missed by conventional cytology, it is limited by a spatial resolution of ~3 μm to 30 μm due to the optical diffraction limit.

Cervical cancer is associated with the persistent infection of high-risk types of human papillomavirus (HPV), together with other socioeconomic co-factors^1^. Screening involves cytological and histological classification of cervical cells. In the UK, cytological examination of cervical squamous cells is classified as normal, borderline or mild dyskaryosis, moderate or severe dyskaryosis and invasive cervical cancer (ICC). Histology is defined as normal, cervical intra-epithelial neoplasia (CIN): CIN1, CIN2, CIN3, or invasive cervical cancer. For atypical cells found in the glandular cells of the cervix, the pre-invasive lesion of adenocarcinoma is defined by changes termed cervical glandular intraepithelial neoplasia (CGIN), and sub-classified as low-grade cervical glandular intra-epithelial neoplasia (LGCGIN) and high-grade cervical glandular intra-epithelial neoplasia (HGCGIN). Squamous and glandular lesions may co-exist together and are defined by the level of CIN together with either LGCGIN or HGCGIN. Conventional screening is flawed as it is dependent on the subjective visual inspection of cytology; this often results in mis-diagnoses when grading samples^2^.

Attenuated total reflection Fourier-transform infrared (ATR-FTIR) spectroscopy has shown potential over conventional screening methods, demonstrating it can segregate grades of cervical cytology more accurately than conventional cytological screening^3^,^4^, classify cervical cytology based on HPV infection with low- or high-risk types^5^, and can diagnose underlying disease more accurately that conventional cytology screening^2^. However, FTIR spectroscopy is limited in spatial resolution by the effect of diffraction, defined as the interference of waves when they hit an obstacle or slit. This effect restricts the spatial resolution of FTIR to about half the wavelength of light or ~3 *μ*m to 30 *μ*m^6^, with the resolution being a measure of how closely the lines of an image can be resolved (*i.e.*, the number of independent pixels per value per unit length).

Scanning near-field optical microscopy (SNOM) belongs to a family of nanoscopic techniques that have shown potential in providing detailed information on cell topography and cytoplasmic structures. SNOM has a clear advantage over conventional infrared (IR) microscopy in terms of spatial resolution because it is able to overcome the diffraction limit; this is achieved by using an apertured fibre optic scanning tip. The SNOM technique requires relatively high photon intensities such as those provided by an IR free electron laser (IR-FEL). The SNOM-IR-FEL enables the simultaneous collection of topography and optical features at scales not normally achieved with conventional IR techniques to produce high quality, chemically-rich images at designated wavelengths with a spatial resolution of ≥0.2 μm^7^,^8^.

Increased synthesis of proteins, lipids and nucleic acids is a condition for the rapid proliferation of cancer cells^9^, and changes in the bioavailability of these biomarkers can reveal important patterns of intracellular change. The IR-FEL on the ALICE accelerator at Daresbury Laboratory (Warrington, UK) is tuneable over the range of 5.5 μm to 8.8 μm (~1818 cm^−1^ to ~1136 cm^−1^), which includes a number of biologically important biomarkers^10^ at designated wavenumbers or wavelengths (Table 1). These biomarkers have previously been used to separate normal, low- and high-grade dyskaryosis and cancer cells from each other^2^.

SNOM has been used to investigate the localisation of molecules within cell membranes of prostate cancer cells^11^. Further research demonstrated SNOM can accurately define both the cell surface and internal structures in both healthy and anomalous sperm, including the acrosome, nucleus and the organisation of mitochondria^12^, and has demonstrated potential for single molecule imaging^13^. The application of SNOM to oesophageal cancer tissue studies provided evidence of its potentiality for cancer diagnosis^8^. The increased spatial resolution of SNOM has the potential to reveal and quantify highly localised cancer-related changes in cervical cells at the sub-cellular level (1–0.1 μm), and more accurately and precisely than conventional IR techniques. The above-described IR-SNOM studies were all carried out in reflection mode. To our knowledge, this is the first publication reporting data obtained using IR-SNOM in transmission mode, and using the technique to image whole cervical cells.

The aim of this study was to assess the potential of SNOM in combination with an IR-FEL in the detection of the biophysical properties of cervical cell abnormalities. Spectra were also collected using traditional ATR-FTIR biospectroscopy to investigate the differences between techniques.

## Results

We recruited 5 patients into this pilot study; the youngest aged 25 years (squamous & glandular pre-invasive lesions [CIN2, HGCGIN]) and the oldest 42 years (squamous lesion; low-grade dyskaryosis). Table 2 shows the characteristics for each patient; limited demography was available for the patient diagnosed with high-grade dyskaryosis.

Two out of the 5 patients were current smokers (high-grade dyskaryosis and CIN2 HGCGIN), and 1 patient was taking antibiotics (CIN2, HGCGIN). Four patients tested positive for HPV; none for HPV 18 and 2 for HPV 16. Both normal and high-grade dyskaryosis tested positive for HPV ‘other’ type (*i.e.*, not high-risk HPV types 16 or 18); only normal had an abnormal high vaginal swab. All patients were tested for bacterial vaginosis and were normal.

**Figure 1:**
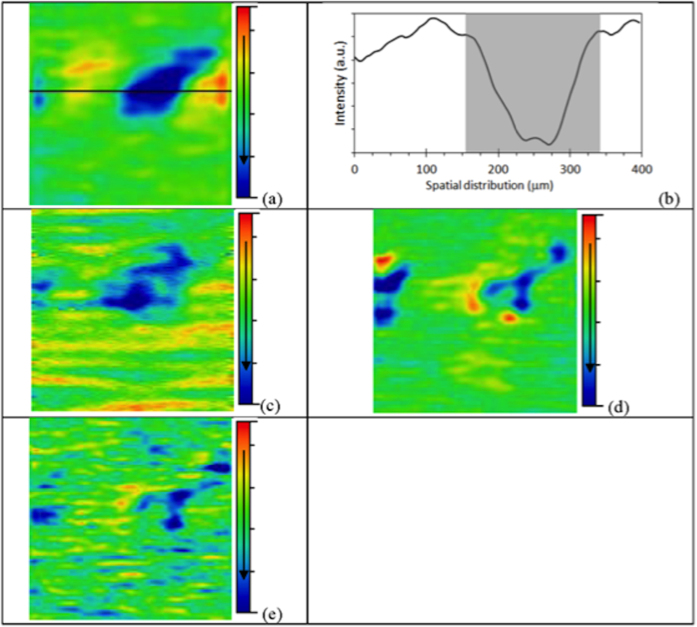
Transmission IR-SNOM images (400 μm × 400 μm) of the same cell sampled from pre-invasive lesion (CIN2, HGCGIN) at the biomarker wavelengths: (**a**) Amide I-6.06 μm (~1650 cm^−1^), the horizontal line in (**a**) is shown in cross-section in (**b,c**) Amide II-6.46 μm (~1550 cm^−1^), (**d**) DNA-8.16 μm (~1225 cm^−1^) and (**e**) Lipids-5.71 μm (1750 cm^−1^). The colour scale bar arrow indicates increasing biomarker absorption. The shaded region in (**b**) corresponds to the interval selected for area calculation according to the cell content. CIN2, HGCGIN: cervical intra-epithelial neoplasia, high-grade cervical glandular intraepithelial neoplasia.

A total of 34 cells were included in the SNOM images. The number of cells was evenly distributed for low-grade dyskaryosis, CIN2, HGCGIN and adenocarcinoma stage 1B1 (6, 5, and 5 cells each, respectively). Sixteen cells were imaged for normal and 2 for high-grade dyskaryosis, which was limited by the number of acceptable cells on the slide. Each SNOM scan comprised topographic, raw transmission (SNOM light) and IR (light) intensity reference images all collected simultaneously at a fixed wavelength. Example SNOM-IR-FEL topography and associated transmission images for the pre-invasive lesion (CIN2, HGCGIN) are presented in Fig. 1 (see Methods: Computational analysis). The topography and associated transmission images for the other 4 cells types is presented in Electronic Supplementary Information (ESI) Figures S1–S4.

### Changes in biomarkers at different grades of cervical dyskaryosis detected by SNOM-IR-FEL

**Figure 2:**
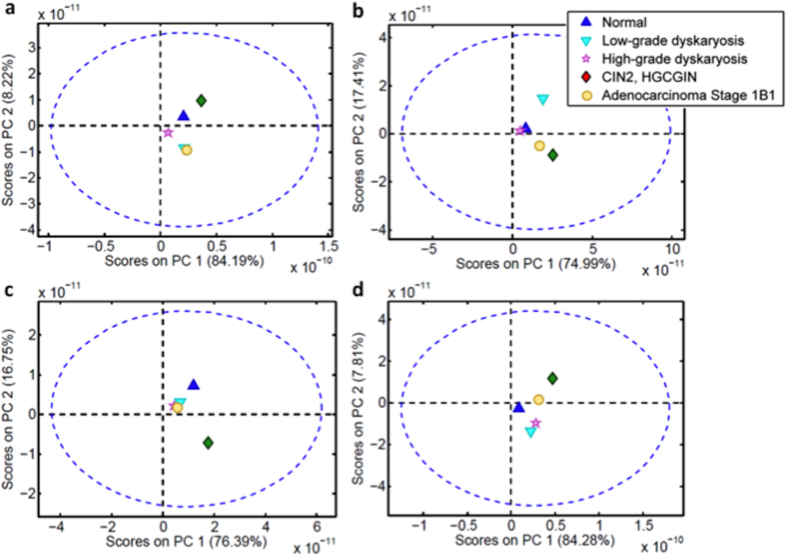
Transmission SNOM-IR-FEL: PC score plots. Scores for 1^st^ and 2^nd^ PCs for the type of cells according to each biomarker response: (**a**) Amide I; (**b**) Amide II; (**c**) Lipids; and, (**d**) DNA. Dotted line indicates 95% confidence limits and shows there were no outliers. CIN2, HGCGIN: cervical intraepithelial neoplasia 2, high-grade cervical glandular intraepithelial neoplasia; PC: principal components; SNOM-IR-FEL: scanning near-field optical microscopy coupled with an infrared-free electron laser.

When applied to spectra, principal component analysis (PCA) identifies common sources of variance across spectra and collates them into a small number of dimensions (*i.e.*, principal components [PCs]). Similar behaviour between samples ‘nests’ them closer together. PC score plots (Fig. 2) show adequate separation for Amide I between normal, high-grade dyskaryosis and CIN2, HGCGIN, with PC1 representing 84.2% and PC2 8.2% of the data variance (Fig. 2a). The overlap observed for low-grade dyskaryosis and adenocarcinoma Stage 1B1 in the Amide I band indicates there was insufficient information to achieve complete class segregation. Clean separation was observed for Amide II between all samples, with the exception of normal and high-grade dyskaryosis, with PC1 and PC2 signifying 74.99% and 17.41%, respectively of the data variance (Fig. 2b). There was considerable overlap for lipids for low- and high-grade dyskaryosis and adenocarcinoma Stage 1B1, with good separation observed between normal and CIN2, HGCGIN; PC1 was 76.39%, and PC2 was 16.75% (Fig. 2c). All five cell types were distinguishable for DNA, although low- and high-grade dyskaryosis were very close to each other, with PC1 representing 84.28% and PC2 7.81% of the data variance (Fig. 2d). Hotelling *T*^*2*^ versus Q Residuals were plotted to assess how well the model described the samples with the optimal score for Hotelling *T*^*2*^ being 100%, and the optimum score for Q Residuals being 0%. These plots show that all the samples fell within the 95% confidence limits and that no outliers were detected (See ESI Figure S5). Validation of the PCA model was performed using Q Residuals to measure variation outside the PCA model for each sample according each biomarker response (see ESI Figure S6); and Hotelling *T*^*2*^ was used to measure variation within the PCA model for each sample according each biomarker response (see ESI Figure S7). All values fell within the 95% confidence limit and show there were no outliers. The *T*^*2*^ and Q residuals plots show the data fits the model well.

**Figure 3:**
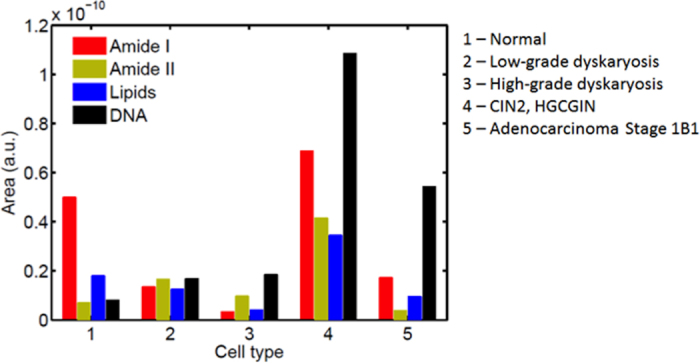
Transmission SNOM-IR-FEL: bar chart for each type of cell according to the biomarker responses. CIN2, HGCGIN: cervical intraepithelial neoplasia 2, high-grade cervical glandular intraepithelial neoplasia; SNOM-IR-FEL: scanning near-field optical microscopy coupled with an infrared-free electron laser.

The area of absorbance for each biomarker for each cell type is shown in Fig. 3, with the percentage of area variation compared with normal shown in Table 3. For low-grade dyskaryosis, Amide I and lipids were lower than normal cells (−73% and −31%, respectively) with large increases detected for Amide II and DNA (143% and 111%, respectively). This pattern of decreased Amide I and lipids was also observed for high-grade dyskaryosis (Amide I: −94%, lipids: −78%), and similarly, Amide II and DNA were higher (40% and 132%, respectively). All four biomarkers were higher for the pre-invasive squamous/glandular cells (CIN2, HGCGIN) than normal cells, with dramatic increases observed for Amide II (509%) and DNA (1272%). Amide I was 38% higher and lipids were 93% higher. Conversely, the profile for adenocarcinoma Stage 1B1 was similar to low-grade dyskaryosis for Amide I (−66%) and lipids (−47%). Adenocarcinoma Stage 1B1 was the only cell type in which a decrease in Amide II was detected (−46%), and DNA availability was approximately half that detected for CIN2, HGCGIN (585%).

**Figure 4:**
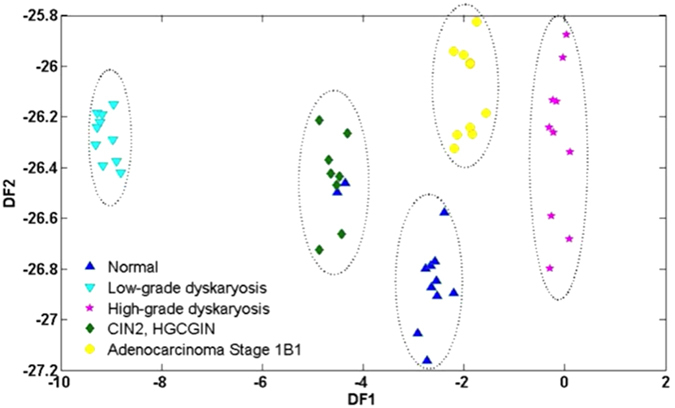
ATR-FTIR Spectroscopy: Discriminant Function (DF) plot for PCA-LDA (6 PCs). This technique promoted a better clustering of the cell types than using PCA alone (See ESI Figure S9). Dotted regions indicate each class. CIN2, HGCGIN: cervical intraepithelial neoplasia 2, high-grade cervical glandular intraepithelial neoplasia; PCA-LDA: principal component analysis coupled to linear discriminant analysis.

Spectra collected by ATR-FTIR spectroscopy for each cell type were very similar to each other, where the signal’s difference appeared to be close to the instrument noise (see ESI Figure S8). However, the application of PCA was able to discriminate each cell type by class (see ESI Figure S9). PCA alone is often not enough to segregate out data classes or clusters sufficiently. By applying a supervised technique such as linear discriminant analysis (LDA) to the PCA output as above (PCA-LDA), it promotes inter-class variation to be identified whilst preventing over-fitting of the data. PCA-LDA revealed good separation of classes, although 2 spectra from the normal set appeared within the CIN2, HGCGIN class (Fig. 4).

**Figure 5:**
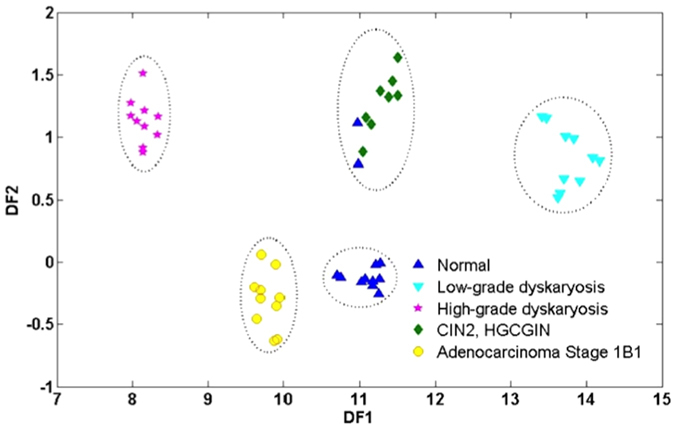
ATR-FTIR spectroscopy: Discriminant Function (DF) plot for SPA-LDA. Using this technique, cells types were clustered more acutely than that observed for PCA-LDA. Dotted regions indicate each class. CIN2, HGCGIN: cervical intraepithelial neoplasia 2, high-grade cervical glandular intraepithelial neoplasia; SPA-LDA: successive projections algorithm in conjunction with linear discriminant analysis.

**Figure 6:**
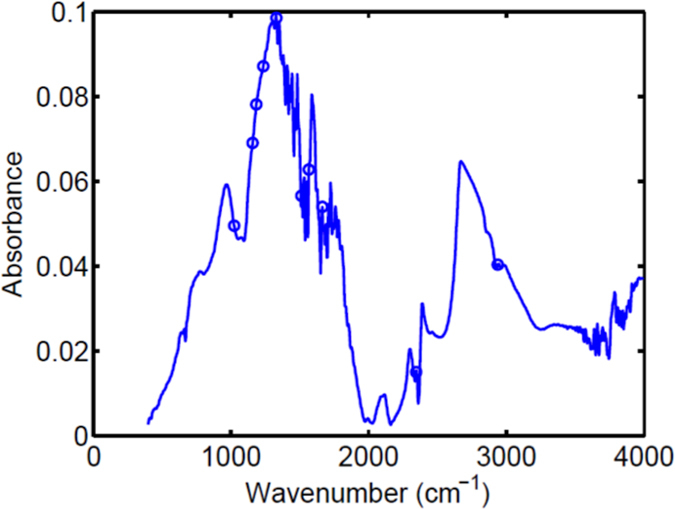
ATR-FTIR spectroscopy: average raw spectrum and its selected variables (circled) by SPA-LDA. SPA-LDA: successive projections algorithm in conjunction with linear discriminant analysis.

Successive projections algorithm (SPA) is a variable selection technique that can produce models with good prediction ability. Used together with LDA (SPA-LDA), this technique produced similar results to PCA-LDA, although the clustering of cell types was more acute (Fig. 5), with discriminant wavenumbers being 1022, 1157, 1184, 1234, 1331, 1512, 1566, 1662, 2345 and 2939 cm^−1^, and 8 of these occupying the fingerprint region (1800-900 cm^−1^); (Fig. 6). The tentative assignment of these wavenumbers in the fingerprint region to associated biomarkers is given in Table 4.

## Discussion

Although the number of cells per patient per cell type is small, the results evidence the promise of the SNOM-IR-FEL technique in obtaining chemical information relevant to the detection of cervical cell abnormalities and cancer diagnosis at high spatial resolution. Clear trends in increased bioavailability of DNA were seen across all four disease cell types compared to normal, with dramatic increases observed for the pre-invasive lesion of squamous/glandular neoplasia and for adenocarcinoma Stage 1B1. Both of these patients were infected with HPV 16, which is known to integrate into the host’s DNA and produce a range of proteins that accelerate biochemical cascades that result in an overexpression of proto-oncogenes, stimulate rapid cell growth and increase the expression of proteins necessary for DNA replication^14^,^15^. It is possible that the increased DNA expressed in these cell types may be a combination of increased human and viral DNA. The dramatic increase in all biomarkers for the pre-invasive squamous/glandular lesion suggests a ‘commitment’ to carcinogenesis, whilst the mechanism behind the down-regulation in proteins and lipids in adenocarcinoma is unclear, and may reflect the tumour achieving some form of steady-state, or that energy supply is exhausted. It should also be noted that ‘normal’ cells were infected with HPV ‘other’ type (*i.e.*, not high-risk HPV type 16 or 18), and this patient had an abnormal high vaginal swab, which may have influenced the profile observed here.

For the squamous lesions (low- and high-grade dyskaryosis), the results suggest the production of lipids and specific proteins featuring Amide I bonding has been down-regulated. An amide bond is formed when two amino acids are joined at the C=O and N-H junction. Amide I is associated with the stretching of C=O bonds in polypeptides, whilst Amide II is associated with the bending of the N-H bond. Previous vibrational work has established correlations between the frequencies of Amides I/II to the secondary structure of polypeptides, which include α-helices, β-sheets, turns and undefined structure^16^. The increases in Amide II for low-grade and high-grade dyskaryosis and the pre-invasive squamous/glandular lesion may be due to the increased production of specific polypeptides that feature Amide II bonding in their secondary structures, and may represent a singular biomarker of importance. Previous work has shown the presence of proteins in the β-sheet conformation has been linked in formation of the protein aggregates and fibrils observed in many human diseases, notably the amyloids seen in Alzheimer’s disease^17^.

Whilst lipids are associated with the proliferation of cancer cells^9^, we detected increases only for the mixed pre-invasive squamous/glandular lesion. However, it should be noted that the wavelength for lipids at 5.71 μm lies near the limit of the IR-FEL where the beam intensity was low and shot-to-shot stability less good than for other wavelengths, which resulted in SNOM transmission images that had a lower signal-to-noise ratio (SNR). Overall, although the SNOM-IR-FEL technique was able to distinguish different cell types according to biomarkers, the delineation was less distinctive between low- and high-grade dyskaryosis than for normal, pre-invasive squamous/glandular lesion and adenocarcinoma Stage 1B1, which may be influenced by the small number of cells imaged. Given that the underlying normal cellular activity of squamous cells is different to mucus-producing glandular cells, it is not unreasonable to assume that the biological processes involved in the development of squamous cervical lesions may be different to glandular lesions.

As expected, spectra collected the traditional way using ATR-FTIR spectroscopy coupled with multivariate analysis, was able to segregate the cell types clearly into classes. Spectra for the normal sample were collected the day before those collected for the pre-invasive squamous/glandular sample; thus the appearance of two spectra within the pre-invasive class is likely due to natural variation of the normal sample rather than cross-contamination. The range of biomarkers available for investigation with ATR-FTIR was broader than that available for the SNOM-IR-FEL measurements in this experiment and encompasses the whole of the fingerprint region. Nonetheless, a large portion of this region was still available for investigation with SNOM-IR-FEL, where the wavelength range is dictated by the ALICE accelerator beam energy and FEL undulator gap settings.

Although SNOM has been shown to reveal cytoplasmic structures in previous studies^12^, the SNOM-IR-FEL images obtained in this study were difficult to interpret in terms of structures. This was due to the use of a cleaved fibre for the SNOM imaging, which added significant tip artefacts to the topographical image and often off-set the topographic from the transmission images. Additionally, the method used to prepare the slides (cytospinning) resulted in many cells rupturing upon impact with the barium fluoride (BaF_2_) slide and left very few whole cells that were free of debris. However, care was taken to avoid any debris within the field of images as much as possible, and to ensure that all images contained the whole cells.

In terms of a diagnostic tool for use in routine patient screening, the technique of ATR-FTIR has low running costs and inexpensive consumables, together with a turnaround of 15–20 minutes per sample. The SNOM-IR-FEL technique has higher running (estimated at £250/hour) and consumables costs, and requires specialised fibre optics that need to be replaced regularly. In this experiment, the time required to collect each SNOM scan, at each wavelength, was approximately 80–100 minutes, depending upon the number of pixels obtained per image. Although building small accelerators on hospital sites is achievable, their installation would require specialised personnel and a safe location away from the main hospital thoroughfare. Therefore, between the two techniques studied here, ATR-FTIR spectroscopy would currently be the technique of choice for routine screening applications.

The results presented here demonstrate that the SNOM-IR-FEL technique is able to correctly identify cervical cell abnormalities using whole cells. In this study, the SNOM technique was used in ‘low resolution’ mode, which enabled direct comparison with the ATR-FTIR method. SNOM has a clear advantage over ATR-FTIR in terms of being a ‘precision tool’ that can be used to identify the location of biomarkers within the cell, leading to further understanding of how cancer develops and in identifying targets of therapeutic potential. It can be tuned to specific wavenumbers/wavelengths, which may help to exclude the ‘noise’ of other biomarkers that lie within close proximity, and makes the extraction of specific biomarkers more accessible.

## Methods

### Study population

Ethical approval was obtained from the National Research Ethics Service Committee London-Fulham (Approval number 13/LO/0126). This study was conducted according to the principles of the Declaration of Helsinki and all other applicable national or local laws and regulations. All patients gave written informed consent before any protocol-specific procedure was performed.

Patients were selected from a cohort of patients taking part in a larger study and were chosen based on their cytology and histology typing (worse grade) to match a diagnosis of ‘normal’, squamous lesions (low-grade dyskaryosis and high-grade dyskaryosis), pre-invasive mixed lesions involving both squamous and glandular cells (CIN2, HGCGIN), or developed glandular lesions (adenocarcinoma). We selected pre-menopausal, non-pregnant women of reproductive age (18–45 years of age) who were scheduled, if necessary, to undergo local cervical treatment at Imperial College NHS Healthcare Trust. All samples were collected prior to treatment. The recruitment commenced in May 2013 and was completed in May 2015.

Patients were anonymous and assigned a unique identifier. We collected patient characteristics that included ethnicity, parity, smoking habits, antibiotic use within the last 2 weeks, phase in their cycle and use of contraception. The type of contraception and the time of their cycle (follicular or luteal) were documented. Medical and gynaecological history was collected for each patient including time since last sexual intercourse. For each patient, we collected data on the cytology, HPV DNA test and typing and histology, if available. Ethnicity was self-reported as Caucasian, Asian or Black.

Women who were HIV or hepatitis B/C positive, women with autoimmune disorders, and women that received pessaries within 14 days of sampling were excluded. Women with a previous history of cervical treatment were also excluded.

### Sample collection

A sterile, disposable speculum was inserted, without lubricant, and a cervical sample of ThinPrep, liquid-based cytology (LBC) was taken from the cervix (ThinPrep, HOLOGIC Inc., Bedford, USA). This was analysed for cytological diagnosis and HPV DNA test and typing. HPV DNA test and 16/18 genotyping was carried out according to manufacturer’s guidelines using the Abbott RealTime High Risk (HR) HPV assay on Abbott M2000 platform; a clinically validated *in vitro* polymerase chain reaction (PCR) assay with identification of HPV-16, -18 and 12 other HR HPV subtypes (31, 33, 35, 39, 45, 51, 52, 56, 58, 59, 66, 68)^18^. From the remaining methanol-based fluid, 1 ml was stored at 4 °C at the Centre for Biophotonics (Lancaster University, England), until preparation for SNOM-IR-FEL or ATR-FTIR analysis.

### Slide preparation

Each sample was agitated to disperse the cell pellet, and a 500-μl aliquot was collected into a clean microtube. The 500-μl aliquots were centrifuged at 2000 rpm for 5 min and the ThinPrep supernatant was aspirated from above the pellet to remove its spectral signature (*i.e.*, the methanol fixative). Each sample was re-suspended in 500 μl of distilled H_2_O, agitated and centrifuged again. The supernatant was removed again and the wash step was repeated once more. For ATR-FTIR spectral analysis, the final pellet was immersed in 100 μl of distilled H_2_O, agitated and dispensed onto IR-reflective glass slides (Low-E; Kevley Technologies Inc., Chesterland, OH, USA) in a uniform spread of whole cells and allowed to bench dry for a minimum of 24 hours. Samples were then stored in a desiccator for a minimum of 48 hours to remove any residual water before spectral analysis.

For SNOM-IR-FEL analysis, a further 500-μl aliquot was washed as described above. If the final pellet was small, it was suspended in 500 μl of distilled H_2_O, and larger pellets in 1000 μl of distilled H_2_O. Each suspension was then agitated to disperse the pellet, and 5–6 drops added to a cytofunnel held in a cytoclip that had been pre-loaded with a BaF_2_ slide (Crystan Ltd, Dorset, UK). Samples were spun at 3000 rpm for 5 min in a Cytospin™ 4 Cytocentrifuge (Thermo Fisher Scientific Inc., MA, USA) to disperse the cells in a single layer on the slide. Slides were then housed in slide cartridges and kept in a desiccator until required.

### SNOM and IR-FEL experimental set-up

The experiments were performed on the IR-FEL beamline at the ALICE energy recovery linear accelerator at Daresbury Laboratory^19^,^20^. The wavelength of light from the FEL was selected by changing the undulator gap and, at the present accelerator settings, could be varied continuously from about 5.5 μm to 8.8 μm (~1818 cm^−1^ to ~1136 cm^−1^), a range which covers a number of biologically important absorption bands. The IR-FEL operates at a macro-pulse repetition rate of 10 Hz, which limits, and determines, the rate of data collection. The IR light from the FEL was transported to the experimental area via an evacuated beamline and exited the beamline through a KBr window. The intensity of the FEL radiation was attenuated using a set of polarisers and focussed onto the sample. A CaF_2_ beam-splitter enables the FEL radiation to be split so that approximately 80% went to the SNOM and 20% was used as a reference signal. The reference signal was monitored with a single-element pyro detector.

The general principle of operation for the SNOM used in these experiments has been previously described^7^. In brief, the scanning tip is a specially prepared IR-transmitting Chalcogenide glass fibre, where one end is etched to a sharp tip. Gold is then evaporated onto the tip so that it covers all but the very end, forming an aperture of 0.1–1 μm in diameter through which the light is collected. The fibre tip is then rastered over the surface of the sample, keeping the tip-to-sample distance constant with shear-force feedback. A single IR-FEL macro-pulse is used for each pixel of the images. The standard mode of operation for IR-SNOM is reflection, where the light approaches the sample at a grazing incidence angle of approximately 15° and the reflected light is collected by the fibre, transmitted through the fibre and detected using a liquid nitrogen cooled mercury-cadmium-telluride (MCT) detector.

Here we report the first measurements made in transmission mode, where the sample was illuminated through the slide; the light that was transmitted through the sample was collected by the fibre. For the measurements reported here, the fibre was cleaved and the entire 6-mm diameter fibre core was used to collect the IR light signal so that a direct comparison could be made with standard IR techniques such as ATR-FTIR. The SNOM was incorporated into an inverted optical microscope, which was used to locate specific cells of interest on the sample and to position them within the SNOM scan area.

A BaF_2_ slide containing the cells was mounted onto the SNOM and scans acquired at fixed wavelengths of 5.71 μm/~1750 cm^−1^ (lipids), 6.06 μm/~1650 cm^−1^ (Amide I), 6.46 μm/~1550 cm^−1^ (Amide II) and 8.16 μm/~1225 cm^−1^ (DNA-asymmetric phosphate stretching vibrations [ν_as_PO_2_^−^]) for each set of cells. Topographic, raw transmission and intensity reference data were collected simultaneously, at a fixed wavelength, for each SNOM image scan.

### Atomic force microscopy (AFM) imaging of cells

To further evidence that whole cervical cells had been used to collect the SNOM-IR-FEL data, atomic force microscopy (AFM) was performed on the adenocarcinoma stage 1B1 sample, using a Bruker Innova AFM in contact mode using silicon nitride probes of nominal spring constant 0.07 N/m (See ESI Figure S10). Topography and deflection (error signal) channels were recorded simultaneously. The contact force of the AFM tip on the cells was minimised to optimise image quality.

### ATR-FTIR spectroscopy

Spectra were acquired using a Tensor 27 FTIR spectrometer with a Helios ATR attachment (Bruker Optik GmbH). Each spectrum comprised 32 scans at 8 cm^−1^ wavenumber spacing with 2× interferogram zero-filling. Before the spectra were taken, the crystal was cleaned with distilled H_2_O and inspected by video camera to be free of any contaminants. A background spectrum was acquired before the sample slide was mounted and the stage moved to bring the cervical cells in contact with the diamond. Spectra were collected from ten random sites on each slide. Spectra were converted to absorbance by Bruker OPUS software (Bruker Inc., Billerica, MA, USA).

### Pre-processing of SNOM-IR-FEL images

The raw forward and backward SNOM transmission images were loaded into the freely available software Gwyddion 2.40, available at http://gwyddion.net/, and converted into text files ready for importing into MATLAB. A second set of raw data files and topographical images were converted into jpgs for image enhancement. No other pre-processing was performed other than file conversion.

### SNOM-IR-FEL image enhancement

The images presented in Fig. 1 and Figures S1–S4 (see ESI) were processed for presentation using Gwyddion 2.40 and a median height line correction in the horizontal (fast scan) axis, followed by the removal of high frequency noise using a two-dimensional Fourier-transform. The computational analysis was performed using raw data.

### Computational analysis

#### SNOM-IR-FEL transmission images

The SNOM-IR-FEL transmission images were processed using MATLAB software 2014a and PLS Toolbox version 7.9.3 (Eigenvector Research, Inc., WA, USA). Each SNOM-IR-FEL data set (transmission images) comprised four matrixes with size of 150 × 150 corresponding to each biomarker response (Fig. 1). To obtain a spectrum-like signal profile from the biomarker response, the biomarker data matrix was converted into a vector by the mean calculation of the matrix in the column-mode direction (Equation 1), where s_j_ is an element of the row-vector s {1 × 150}, corresponding to the spectrum-like signal; *m* is the size of the image on column-mode direction; and x_ij_ is an element of the biomarker matrix X.


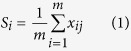


Thereafter, the spectrum-like signal was normalized by mean-centring and absolute value. The bar charts were made with the area of the spectrum-like signal integrated into an interval of spatial distribution according to the cell content position (Fig. 1).

PCA was performed with the whole spectrum-like signal using only mean-centring and absolute value as pre-processing. A summary of the computational steps in processing the data is given in Figure S11 (see ESI). PCA is an unsupervised technique commonly used as the first step in analysing large, multivariate datasets. Unsupervised techniques require no information from the user but rely instead on an internal criterion to guide learning. In unsupervised learning, the system forms clusters (groupings, regions of data space). In general terms, PCA reduces the dimensionality of large datasets and using mathematical projection, the original data set which may have involved many variables, can often be interpreted in just a few variables (the principal components; PCs). This reduced dimensional data set will allow the user to spot trends, patterns and outliers in the data, far more easily than would have been possible without performing the PCA. When applied to spectra, PCA identifies common sources of variance across spectra and collates them into a small number of dimensions. PCA is often not enough to segregate out data classes or clusters sufficiently. By applying a supervised technique such as LDA to the PCA output, it promotes inter-class variation to be identified whilst preventing over-fitting of the data.

PCA was executed using the average signal of each biomarker (triplicate) for five samples, one for each type of cell morphology: normal, low-grade dyskaryosis, high-grade dyskaryosis, CIN2, HGCGIN and adenocarcinoma Stage 1B1. Additionally, the area for each biomarker for each cell type was determined, as was the percentage area variation from ‘normal’ for each biomarker for each cell type.

#### ATR-FTIR spectra

The ATR-FTIR data were analysed using multivariate techniques of PCA for preliminary data reduction, and the output was processed using LDA and a variable selection technique employing SPA^21^, in conjunction with LDA for selecting an appropriate subset of wavenumbers for classification purposes. SPA is a variable selection technique specifically designed to improve the conditioning of multiple linear regression by minimizing collinearity effects in the calibration dataset and can result in models with good prediction ability^22^.

The classic Kennard–Stone (KS) uniform sampling algorithm^23^ was adopted to divide the available samples into training (70%), validation (15%) and prediction sets (15%) for construction and validation of the PCA-LDA and SPA-LDA models. The training set was used to obtain model parameters (including variable selection for LDA), and the validation set was employed to choose the best number of the PCs for PCA model and to guide the variable selection. The optimum number of variables for SPA–LDA was used to select variables employing the G function as cost function^23^.

## Additional Information

**How to cite this article**: Halliwell, D. E. *et al.* Imaging cervical cytology with scanning near-field optical microscopy (SNOM) coupled with an IR-FEL. *Sci. Rep.* **6**, 29494; doi: 10.1038/srep29494 (2016).

## Supplementary Material

Supplementary Information

## Figures and Tables

**Table 1:** Important biomarkers (wavenumbers and associated wavelength).

Wavenumber (cm^−1^) and associated biomarker	Wavelength (μm)
~1225 (DNA – asymmetric phosphate stretching vibrations)^a^	8.16
~1650 (Amide I of proteins predominantly in α-helix conformation)^a^	6.06
~1550 (Amide II of proteins predominantly in *β-*sheet conformation)^a^	6.46
~1750 (Lipids)^a^	5.71

^a^Movasaghi *et al.*^10^; N.B.: The signal at a particular wavenumber could have contributions from more than one biomarker. Amide I and II are linked to the secondary structure of proteins and are indicative of their bioavailability.

**Table 2:** Patient characteristics.

Characteristics	Normal (n = 1)	Low-grade dyskaryosis (Squamous) (n = 1)	High-grade dyskaryosis^a^ (Squamous) (n = 1)	CIN2, HGCGIN Pre-invasive (Squamous & glandular) (n = 1)	Adenocarcinoma Stage 1B1 (Glandular) (n = 1)
No cells imaged	16	6	2	5	5
Age	31	42	Unknown	25	36
Ethnicity	Caucasian	Caucasian	Unknown	Caucasian	Caucasian
Smoker	No	No	Yes	Yes	No
Co-morbidities	Reflux	No	No	Acne	No
Current medications	Omeprazole	Nil	Unknown	Erythromycin	Nil
Recent antibiotics (within last 2 weeks)	No	No	No	Yes	No
Recent pessaries	No	No	No	No	No
Contraception	Nil	Copper IUD	COCP	COCP	Condoms
48 hours since last intercourse	Yes	Yes	Unknown	Yes	Yes
Phase of menstrual cycle	Follicular	Follicular	Luteal	Luteal	Unknown
Vaginal pH	4.4	5	Unknown	4.4	4.4
Mid-stream specimen of urine	Mixed growth	Mixed growth	No growth	No growth	No growth
High vaginal swab	Abnormal	Normal	Normal	Normal	Normal
Cytology, histology, HPV					
Referral smear	Negative	Moderate	Unknown	Severe	Severe
Biopsy	NA	CIN1	CIN2	Micro-invasive SMILE	HGCGIN
Cone	NA	HPV	CIN3	CIN2, HGCGIN	Adenocarcinoma Stage 1B1
HPV positive test	Positive	Unknown	Positive	Positive	Positive
HPV 18	No	Unknown	No	No	No
HPV 16	No	Unknown	Unknown	Yes	Yes
HPV other type	Yes	Unknown	Yes	No	No

^a^There was limited data available for the patient diagnosed with high-grade dyskaryosis. COCP: Combined oral contraceptive pill; HPV: Human Papillomavirus; CIN2, HGCGIN: Cervical intraepithelial neoplasia 2, high-grade cervical glandular intraepithelial neoplasia; IUD: Intrauterine device; POP: Progesterone-only pill; NA: Not applicable; SMILE: Stratified Mucinous Intraepithelial Lesion.

**Table 3:** Percentage of area variation (ΔA (%)) from the ‘normal’ cell morphology for each biomarker for the type of cell.

Cell type	ΔA (%)			
	Amide I	Amide II	Lipids	DNA
Normal	–	–	–	–
Low-grade dyskaryosis	−73	143	−31	111
High-grade dyskaryosis	−94	40	−78	132
CIN2, HGCGIN	38	509	93	1272
Adenocarcinoma Stage 1B1	−66	−46	−47	585

CIN2, HGCGIN: Cervical intraepithelial neoplasia 2, high-grade cervical glandular intraepithelial neoplasia.

**Table 4:** Tentative assignment of biomarkers to wavenumbers.

Wavenumber (cm^−1^)	Corresponding wavelength (μm)	Tentative assignment of biomarkers^a^
~1022	9.8	Glycogen
~1157	8.6	C-O Proteins and carbohydrates
~1184	8.4	Amide III; deoxyribose
~1234	8.1	Amide III as well as phosphate
~1331	7.5	stretching vibrations of nucleic acids
~1512	6.6	Polysaccharides; collagen
~1566	6.4	Amide II
~1662	6.02	Amide I; ring base

^a^Movasaghi *et al.*, 2008 10; N.B.: The signal at a particular wavenumber could have contributions from more than one biomarker.

## References

[b1] Castellsagué X., Bosch F. X. & Muñoz N. Environmental co-factors in HPV carcinogenesis. Virus Res. 89, 191–199 (2002). 12445659 10.1016/s0168-1702(02)00188-0

[b2] Gajjar K. *et al.* Histology verification demonstrates that biospectroscopy analysis of cervical cytology identifies underlying disease more accurately than conventional screening: removing the confounder of discordance. PLoS One 9, e82416 (2014). 24404130 10.1371/journal.pone.0082416 PMC3880266

[b3] Purandare N. C. *et al.* Infrared spectroscopy with multivariate analysis segregates low-grade cervical cytology based on the likelihood to regress, remain static or progress. Anal. Methods 6, 4576–4584 (2014).

[b4] Purandare N. C. *et al.* Biospectroscopy insights into the multi-stage process of cervical cancer development: probing for spectral biomarkers in cytology to distinguish grades. Analyst 138, 3909–3916 (2013). 23338619 10.1039/c3an36527a

[b5] Lima K. M. G. *et al.* Classification of cervical cytology for human papilloma virus (HPV) infection using biospectroscopy and variable selection techniques. Anal. Methods 6, 9643–9652 (2014).

[b6] Harrison A. J., Bilgili E. A., Beaudoin S. P. & Taylor L. S. Atomic force microscope infrared spectroscopy of griseofulvin nanocrystals. Anal. Chem. 85, 11449–11455 (2013). 24171582 10.1021/ac4025889 PMC3889117

[b7] Cricenti A. *et al.* Very high resolution near-field chemical imaging using an infrared free electron laser. Phys. Chem. Chem. Chem. 4, 2738–2741 (2002).

[b8] Smith A. D. *et al.* Near-field optical microscopy with an infra-red free electron laser applied to cancer diagnosis. Appl. Phys. Lett. 102, 053701 (2013).

[b9] Baenke F., Peck B., Miess H. & Schulze A. Hooked on fat: the role of lipid synthesis in cancer metabolism and tumour development. Dis. Model Mech. 6, 1353–1363 (2013). 24203995 10.1242/dmm.011338 PMC3820259

[b10] Movasaghi Z., Rehman S. & ur Rehman I. Fourier transform infrared (FTIR) spectroscopy of biological materials. Appl. Spectosc. Rev. 43, 134–179 (2008).

[b11] Walker K.-A. D., Morgan C., Doak S. H. & Dunstan P. R. Quantum dots for multiplexed detection and characterisation of prostate cancer cells using a scanning near-field optical microscope. PLoS One 7, e31592 (2012). 22347497 10.1371/journal.pone.0031592 PMC3276582

[b12] Andolfi L. *et al.* The application of scanning near field optical imaging to the study of human sperm morphology. J Nanobiotechnology. 13, 2 (2015). 25591971 10.1186/s12951-014-0061-5 PMC4302611

[b13] Zhong L., Wentao L., Wang X. & Cai J. Detection the specific marker of CD3 molecules of human peripheral T lymphocytes using SNOM and quantum dots. Colloids and Surfaces A: Physiochem. Eng. Aspects 313–314, 642–646 (2008).

[b14] Tsai T.-C. & Chen S.-L. The biochemical and biological functions of human papillomavirus type 16 E5 protein. Arch. Virol. 148, 1445–1453 (2003). 12898324 10.1007/s00705-003-0111-z

[b15] Munger K. *et al.* Mechanisms of human papillomavirus-induced oncogenesis. Viro. 78, 11451–11460 (2004). 10.1128/JVI.78.21.11451-11460.2004 PMC523272 15479788

[b16] Krimm S. Jr. & Reisdorf W. C. Understanding normal modes of proteins. Faraday Discuss. 99, 181–197 (1994). 10.1039/fd9949900181 27897017

[b17] Nelson R. *et al.* Structure of the cross-beta spine of amyloid-like fibrils. Nature 435, 773–778 (2005). 15944695 10.1038/nature03680 PMC1479801

[b18] Coutlée F., Rouleau D., Ferenczy A. & Franco E. The laboratory diagnosis of genital human papillomavirus infections. Can J Infect Dis Med Microbiol. 16, 83–91 (2005). 18159534 10.1155/2005/798710 PMC2095016

[b19] Thompson N. R. *et al.* First lasing of the ALICE infra-red free-electron laser. *Nucl. Instrum. Methods Phys. Res.* A 680, 117–123 (2012).

[b20] Thompson N. R. *et al.* Status of the ALICE IR-FEL: from ERL demonstrator to user facility. *International Free Electron Laser Conference-FEL 2015*, Daejeon, Korea, 2014: TUP015.

[b21] Theophilou G., Lima K. M. G., Martin-Hirsch P. L., Stringfellow H. F. & Martin F. L. ATR-FTIR spectroscopy coupled with chemometric analysis discriminates normal, borderline and malignant ovarian tissue: classifying subtypes of human cancer. Analyst 141, 585–594 (2016). 26090781 10.1039/c5an00939a

[b22] Galvão R. K. H., Araújo M. C. U., Silva E. C., José G. E., Soares S. F. C. & Paiva H. M. Cross-validation for the selection of spectral variables using the successive projections algorithm. J. Braz. Chem. Soc. 18, 1580–1584 (2007).

[b23] Kennard R. W. & Stone L. A. Computer aided design of experiments. Technometrics 11, 137–148 (1969).

