# Supplementary material for: Imaging cervical cytology with scanning near-field optical microscopy (SNOM) coupled with an IR-FEL
Source: Sci Rep. 2016 Jul 12;6:29494. doi: 10.1038/srep29494 (PMC4942606; doi:10.1038/srep29494)
Supplement: Supplementary Information [file srep29494-s1.pdf]

# Electronic Supplementary Information

**Imaging cervical cytology with scanning near-field optical microscopy (SNOM) coupled with an IR-FEL**

Diane E. Halliwell,[a] Camilo L. M. Morais,[b] Kássio M. G. Lima,[b] Júlio Trevisan,[c] Michele R. F. Siggel-King,[de] Tim Craig,[d] James Ingham,[d] David S. Martin,[d] Kelly A. Heys,[a] Maria Kyrgiou,[f,g] Anita Mitra,[f,g] Evangelos Paraskevaidis,[h] Georgios Theophilou,[i] Pierre L. Martin-Hirsch,[aj] Antonio Cricenti,[k] Marco Luce,[k] Peter Weightman,[d] and Francis L. Martin[a,l]

*[a]Centre for Biophotonics, LEC, Lancaster University, Lancaster, UK; [b]Biological Chemistry and Chemometrics, Institute of Chemistry, Federal University of Rio Grande do Norte, Natal 59072-970, RN, Brazil; [c]Institute of Astronomy, Geophysics and Atmospheric Sciences, University of São Paulo, Brazil; [d]Department of Physics, University of Liverpool, Oliver Lodge Building, Liverpool, UK; [e]Accelerator Science and Technology Centre (ASTEC), STFC Daresbury Laboratory, UK; [f]Institute of Reproductive and Developmental Biology, Department of Surgery & Cancer, Faculty of Medicine, Imperial College, London, UK; [g]West London Gynaecological Cancer Centre, Imperial College NHS Healthcare, London, UK; [h]Department of Obstetrics and Gynaecology, University of Ioannina, Ioannina, Greece; [i]St James Hospital, Leeds, West Yorkshire, UK; [j]Department of Obstetrics and Gynaecology, Lancashire Teaching Hospitals NHS Trust Foundation, Preston, UK; [k]Istituto di Struttura della Materia, CNR, via del Fosso del Cavaliere 100, Rome, Italy; [l]School of Pharmacy and Biomedical Sciences, University of Central Lancashire, Preston, UK*

Number of Pages = 13
Number of Figures = 11

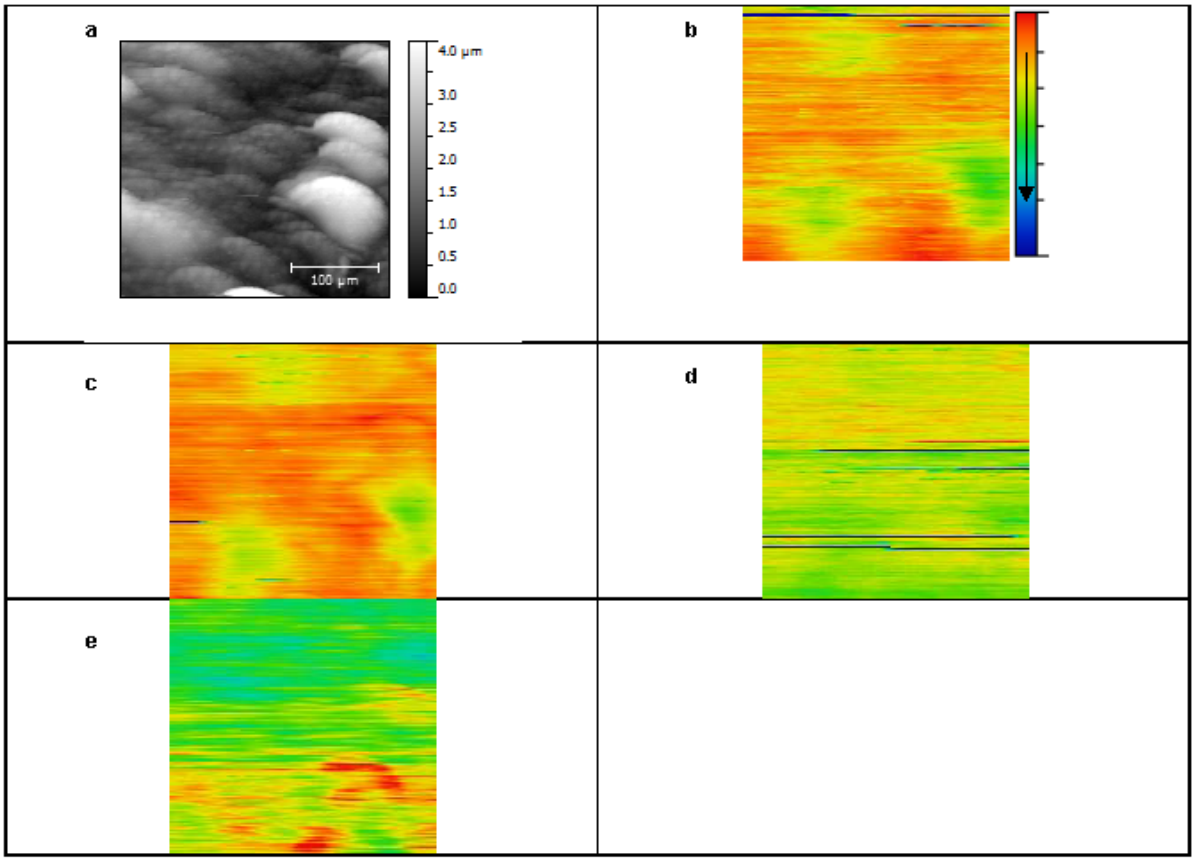


**Figure S1**. **SNOM-IR-FEL images of normal cells:** (**a**) topography; **transmission images:** (**b**) Amide I; (**c**) Amide II; (**d**) Lipids; and, (**e**) DNA. The colour scale bar arrow in (**b**) applies to (**b-e**) and indicates increasing biomarker absorption. SNOM-IR-FEL: Scanning near-field optical microscopy coupled with an infrared-free electron laser.

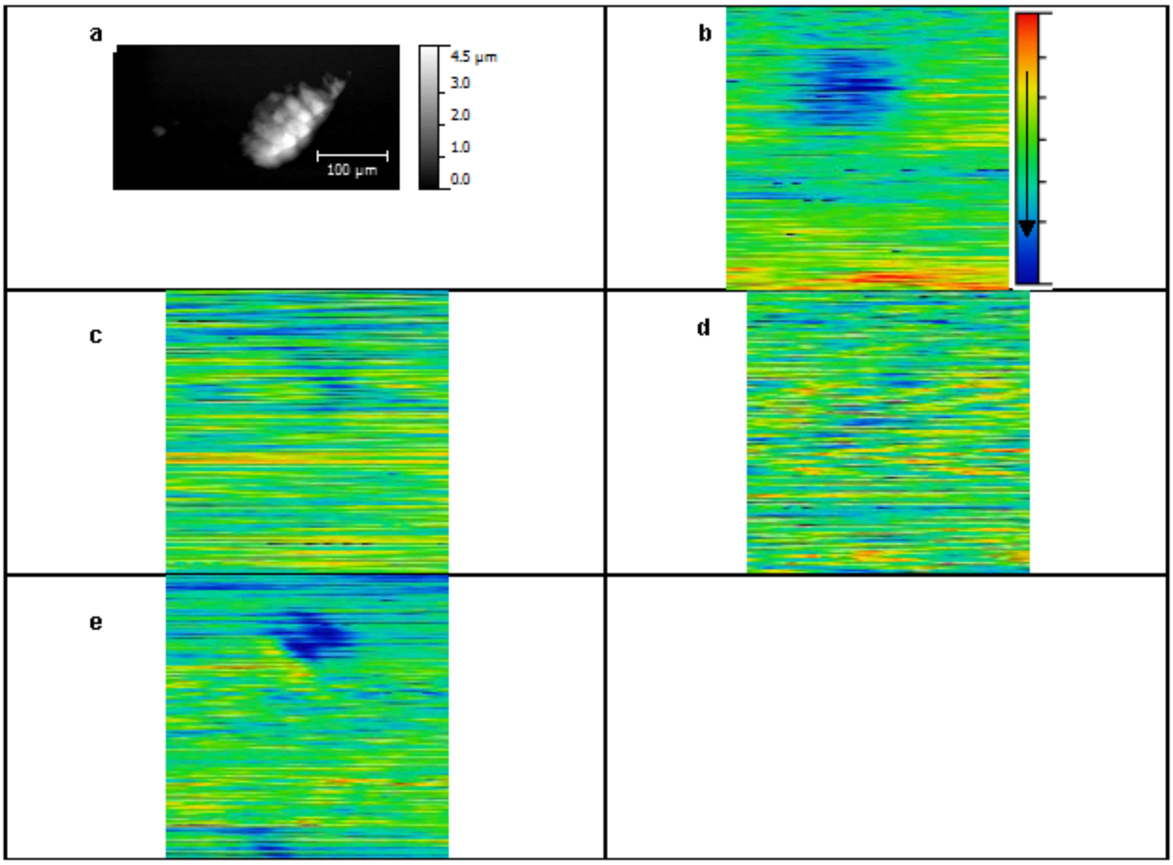


**Figure S2**. **SNOM-IR-FEL images of low-grade dyskaryosis:** (**a**) topography; **transmission images:** (**b**) Amide I; (**c**) Amide II; (**d**) Lipids; and, (**e**) DNA. The colour scale bar arrow in (**b**) applies to (**b-e**) and indicates increasing biomarker absorption. SNOM-IR-FEL: Scanning near-field optical microscopy coupled with an infrared-free electron laser.

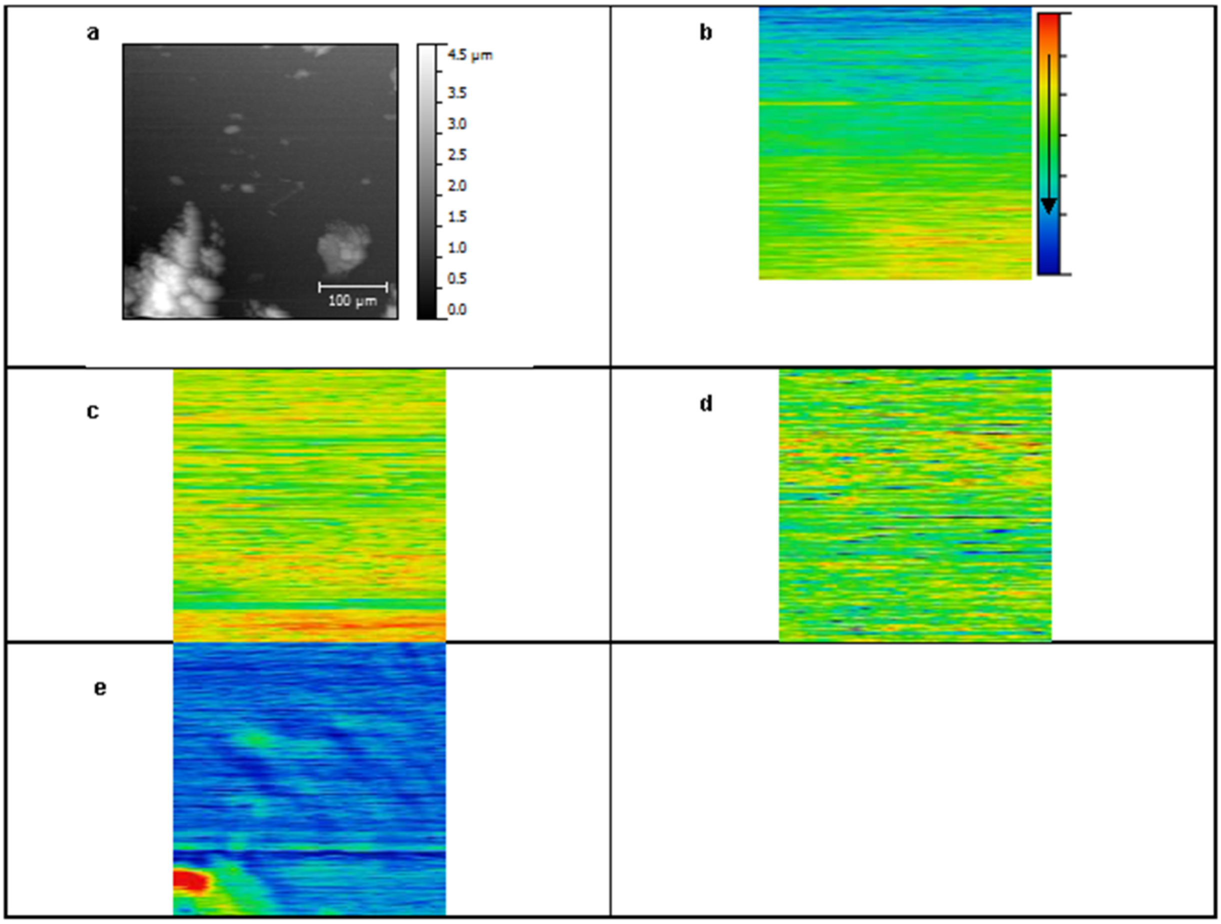


**Figure S3**. **SNOM-IR-FEL images of high-grade dyskaryosis**: (**a**) topography; **transmission images:** (**b**) Amide I; (**c**) Amide II; (**d**) Lipids; and, (**e**) DNA. The colour scale bar arrow in (**b**) applies to (**b-e**) and indicates increasing biomarker absorption. SNOM-IR-FEL: Scanning near-field optical microscopy coupled with an infrared-free electron laser.

The SNOM-IR-FEL images and associated topography of the pre-invasive lesion (CIN2, HGCGIN) are presented in the main body of the text (see Figure 6).

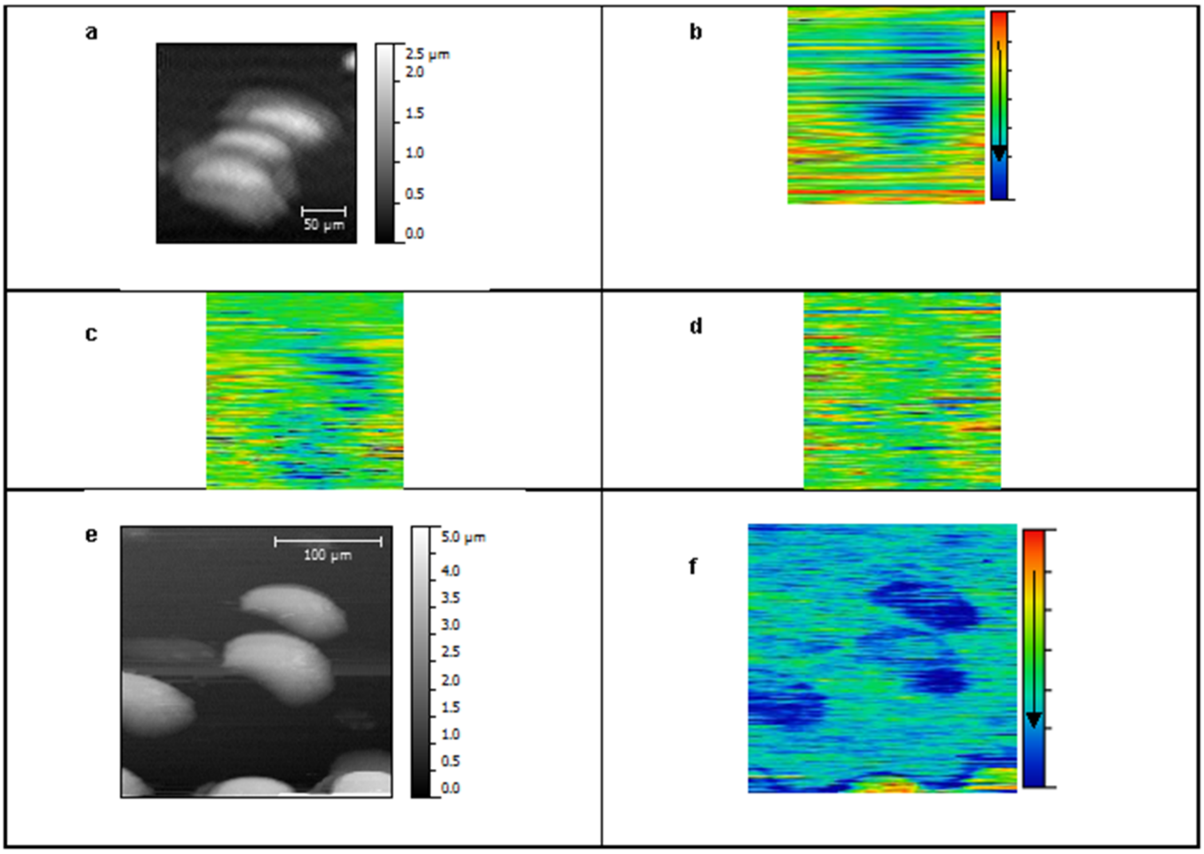


**Figure S4**. **SNOM-IR-FEL images of adenocarcinoma Stage 1B1:** (**a**) topography; **transmission images:** (**b**) Amide I (imaged from different site to topography shown here); (**c**) Amide II; and, (**d**) Lipids. (**e**) Topography of cells from a second area; and, (**f**) the corresponding SNOM transmission image for the DNA biomarker. The colour scale bar arrow in (**b**) applies to (**b-d, f**) and indicates increasing biomarker absorption. SNOM-IR-FEL: Scanning near-field optical microscopy coupled to an infrared-free electron laser.

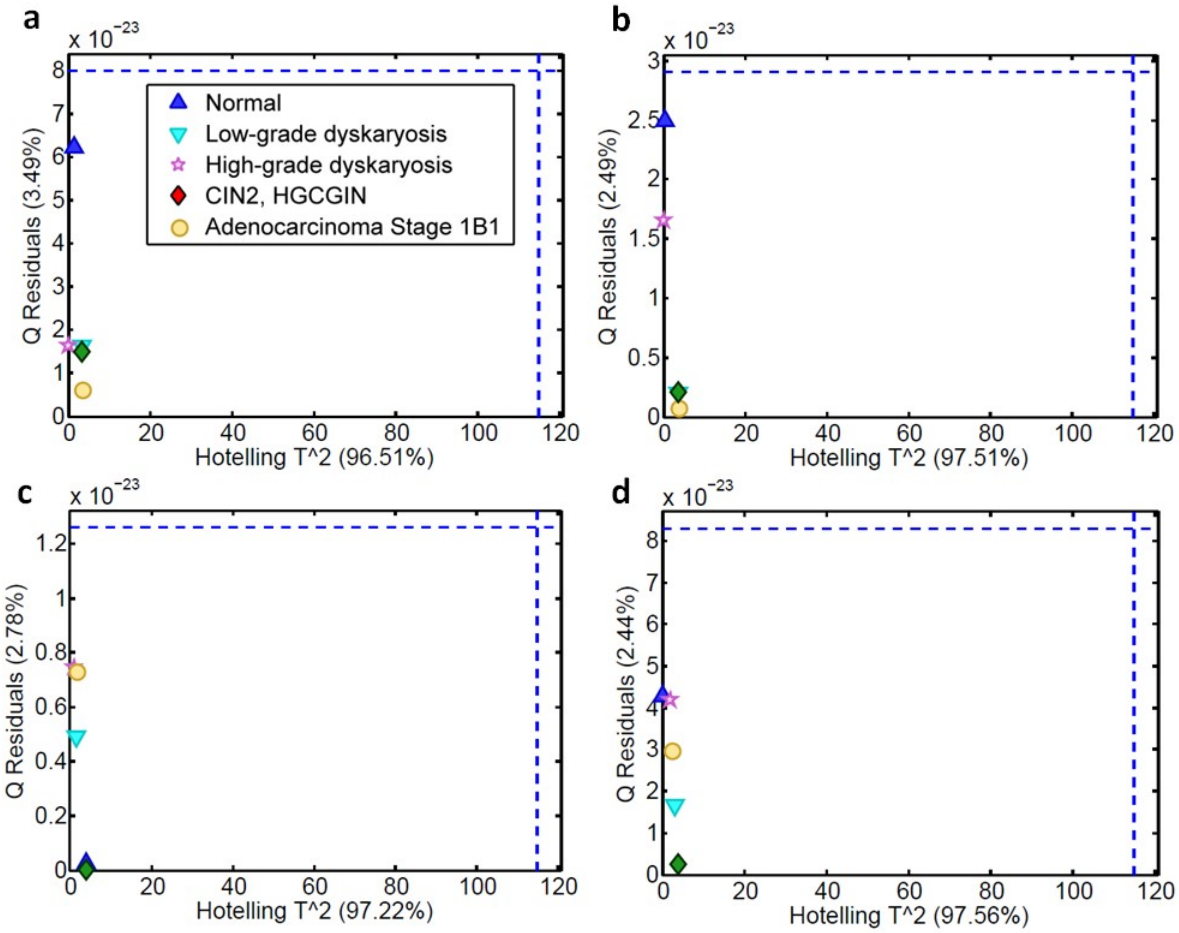


**Figure S5. Transmission SNOM-IR-FEL:** Hotelling $T^2$ versus Q Residuals graphs for the type of cells according to each biomarker response: (**a**) Amide I; (**b**) Amide II; (**c**) Lipids; and, (**d**) DNA. All 5 samples fell within the 95% confidence limits (blue dotted line), and shows there were no outliers. The score for Hotelling $T^2$ ranged from 96.51% to 97.56%; whilst the score for Q residuals ranged from 2.44% and 3.49%. CIN2, HGCGIN: Cervical intraepithelial neoplasia 2, high-grade cervical glandular intraepithelial neoplasia; SNOM-IR-FEL: Scanning near-field optical microscopy coupled with an infrared-free electron laser.

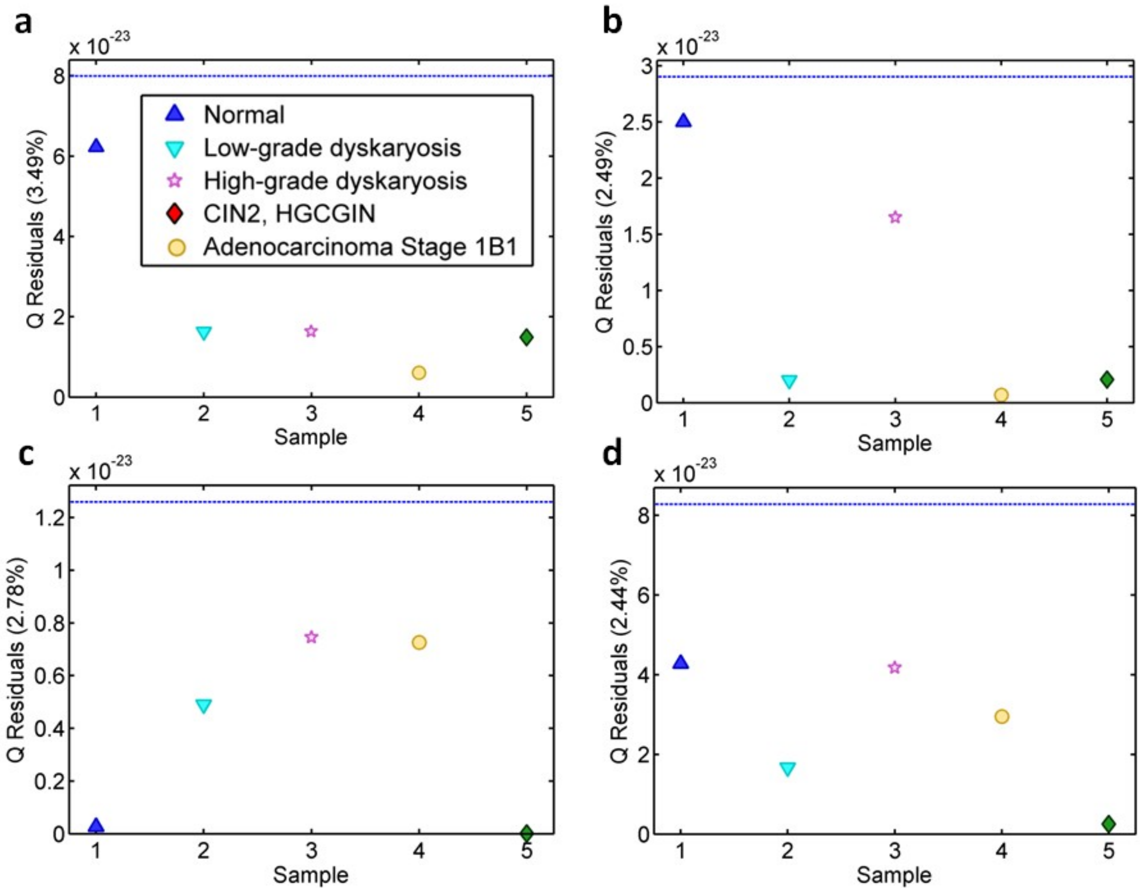


**Figure S6. Transmission SNOM-IR-FEL:** Validation of the PCA model using Q Residuals to measure variation outside the PCA model for each sample according each biomarker response: (**a**) Amide I; (**b**) Amide II; (**c**) Lipids; and, (**d**) DNA. The optimal score for Q Residuals is 0% and here ranged from 2.44% to 3.49%. All 5 samples fell within the 95% confidence limits (blue dotted line), shows there were no outliers and that the data fits the model well. CIN2, HGCGIN: Cervical intraepithelial neoplasia 2, high-grade cervical glandular intraepithelial neoplasia; SNOM-IR-FEL: Scanning near-field optical microscopy coupled with an infrared-free electron laser.

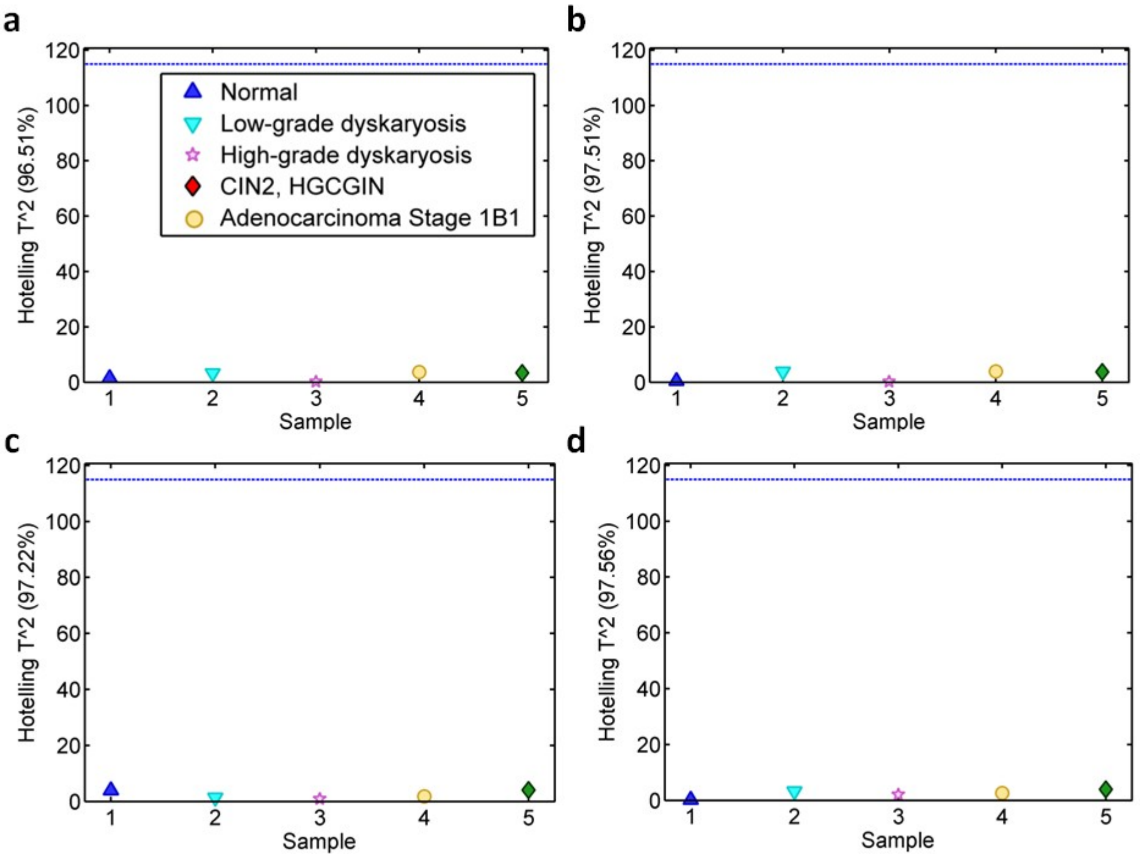


**Figure S7**. **Transmission SNOM-IR-FEL:** Validation of the PCA model using Hotelling $T^2$ to measure variation within the PCA model for each sample according each biomarker response: (**a**) Amide I; (**b**) Amide II; (**c**) Lipids; and, (**d**) DNA. The optimal score for Hotelling $T^2$ is 100% and here ranged from 96.51% to 97.56%. All 5 samples fell within the 95% confidence limits (blue dotted line), shows there were no outliers and that the data fits the model well. CIN2, HGCGIN: Cervical intraepithelial neoplasia 2, high-grade cervical glandular intraepithelial neoplasia; SNOM-IR-FEL: Scanning near-field optical microscopy coupled with an infrared-free electron laser.

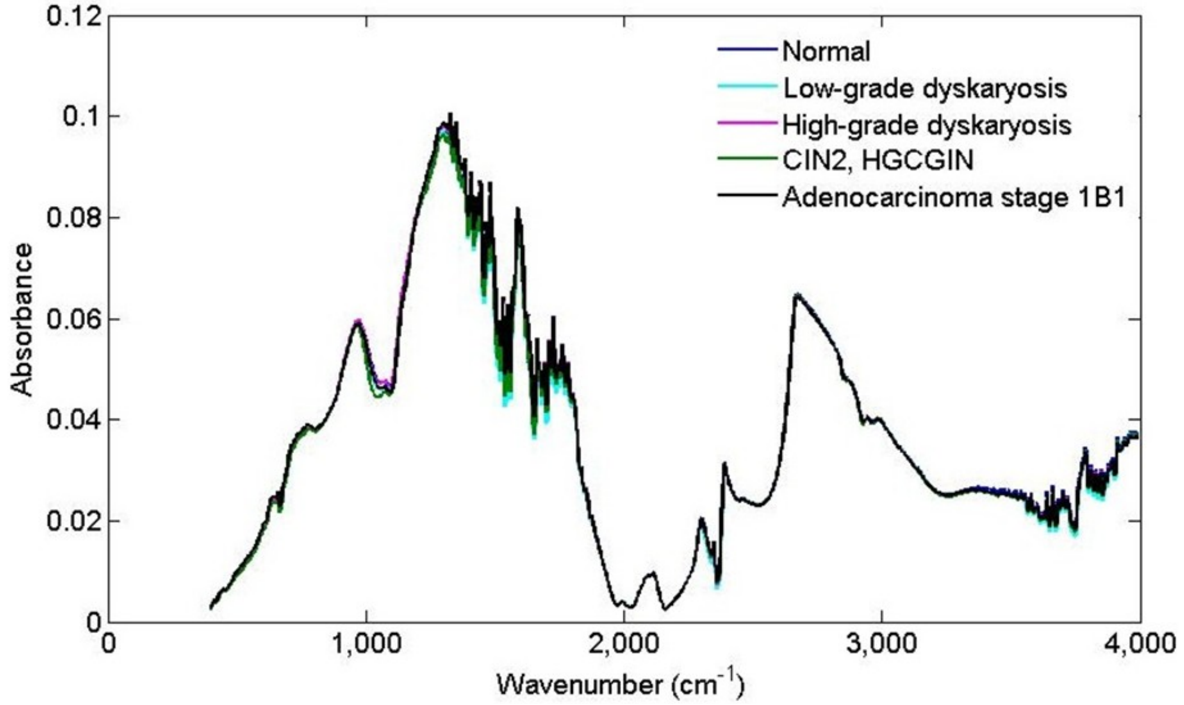


**Figure S8: ATR-FTIR spectroscopy:** Average infrared spectra of cell types.

ATR-FTIR spectroscopy: Attenuated total reflection Fourier-transform infrared spectroscopy; CIN2, HGCGIN: Cervical intraepithelial neoplasia 2, high-grade cervical glandular intraepithelial neoplasia.

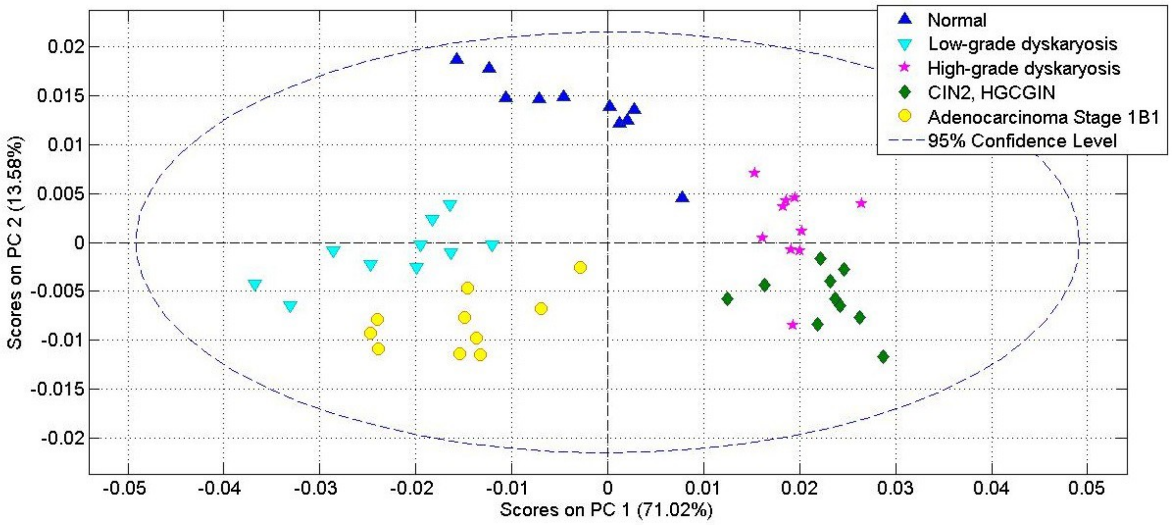


**Figure S9**. **ATR-FTIR spectroscopy:** Scores plot of 1st and 2nd principal components at a 95% confidence level. ATR-FTIR spectroscopy: Attenuated total reflection Fourier-transform infrared spectroscopy; CIN2, HGCGIN: Cervical intraepithelial neoplasia 2, high-grade cervical glandular intraepithelial neoplasia; principal components.

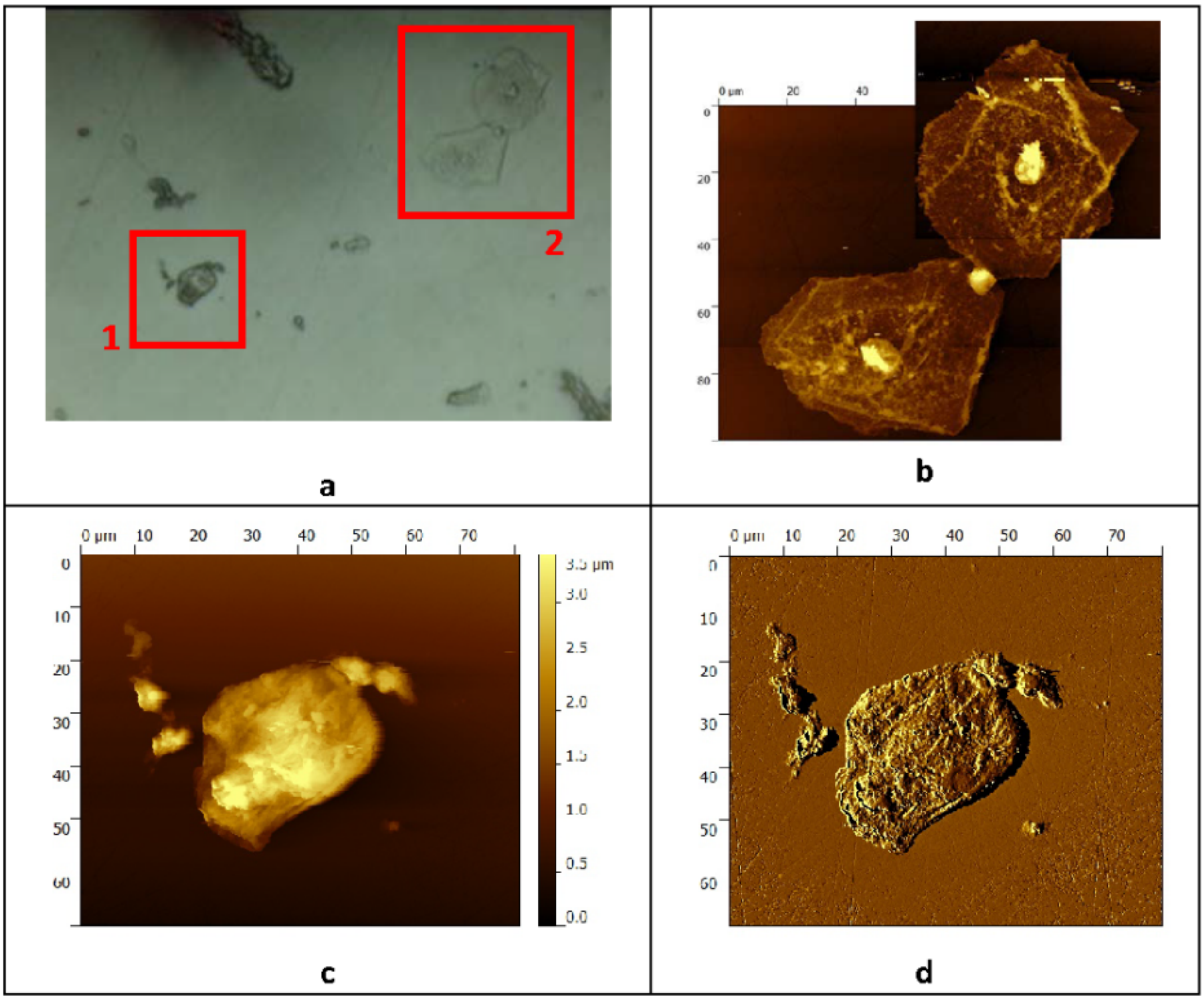


**Figure S10. AFM imaging of adenocarcinoma Stage 1B1:** (**a**) Optical image (×10 magnification) identifying cells for investigation by AFM; and, (**b**) AFM topography image of two intermediate glandular cells [area 2 in (**a**)], the lower cell has two nuclei. The cells exhibit a long axis of ~75 µm. The cell thickness was measured at ~200 nm, whereas the nuclei protruded ~1 µm in height from the substrate. (**c**) AFM topography; and, (**d**) deflection image of a cell identified [area 1 in (**a**)] as having a single enlarged nucleus separated from the rest of the cell by a halo. AFM: atomic force microscopy.

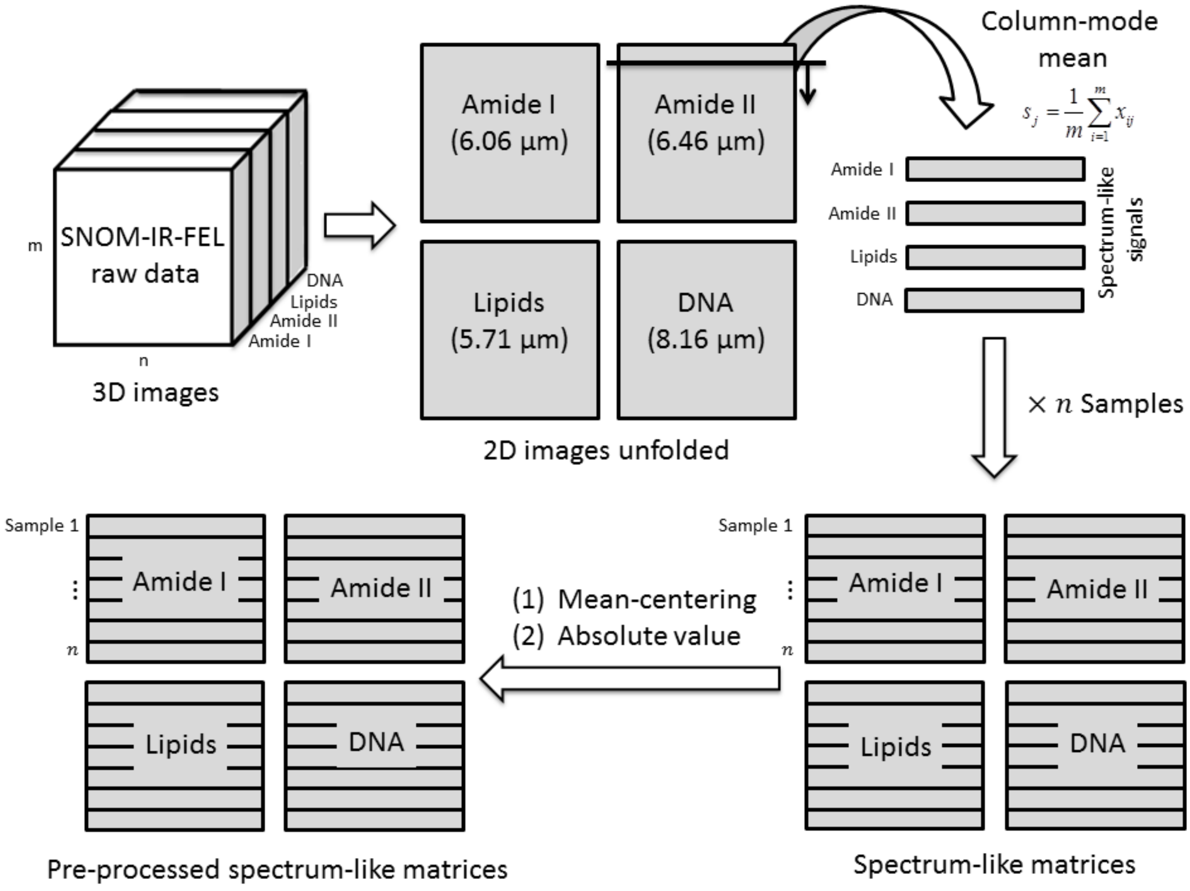


**Figure S11.** The computational steps taken in processing the data.
